# Unsupervised clustering FLIM-phasor from multifunctionalized nanoparticles in living cancer cells

**DOI:** 10.1088/2050-6120/ae7fed

**Published:** 2026-07-01

**Authors:** Dora-Luz Flores, Esperanza Guerra-Rosas, Balam Benítez-Mata, Gustavo Hirata, Michelle Digman

**Affiliations:** 1 Facultad de Ingeniería, Arquitectura y Diseño, Universidad Autónoma de Baja California, Ensenada, Baja California 22860, Mexico; 2 Centro de Nanociencias y Nanotecnología, Ensenada, Baja California 22860, Mexico; 3 Department of Biomedical Engineering, University of California Irvine, Irvine, CA 92697, United States of America; 4 These authors contributed equally to this work.

**Keywords:** segmentation, clustering, FLIM, cancer cells, multifunctionalized nanoparticles

## Abstract

In the present work, we developed a data-driven algorithm for the automated segmentation of fluorescence lifetime imaging microscopy (FLIM) images, enhancing the analysis of multifunctionalized nanoparticles (NPs) within living cancer cells. FLIM, a powerful microscopy technique, generates images that capture the fluorescence lifetime across a sample at the pixel level, revealing critical details about the molecular environment. Traditionally, FLIM image analysis has relied on manual segmentation with the phasor plot approach, a graphical representation of FLIM data in a *G* and *S* coordinate system, which is susceptible to user bias and inconsistency. Here, we present an automated and free of user-biased thresholding and segmentation algorithm that streamlines with clustering techniques to automatically identify phasor-clusters in the phasor plot space, reducing user dependency and providing a reproducible strategy under tested conditions image segmentation. We demonstrate its application in the context of FLIM images displaying a map of intensity heterogeneity where functionalized NPs affect the cellular metabolism of HeLa cells, reported by NADH, a bright and a dim fluorescent source, respectively, both of biological relevance. This algorithm provides a transparent and reproducible approach for FLIM image analysis, showing good agreement with expert-defined segmentation under sufficient contrast conditions, while presenting limitations in low-contrast or noisy regimes.

## 1. Introduction

The average time a fluorophore spends in the excited state before emitting a photon and returning to the ground state is called fluorescence lifetime, a process highly sensitive to its environment such as changes in pH and ion concentration. In addition, nicotinamide adenine dinucleotide in its reduced form (NADH) is an endogenous fluorescent molecule widely used to monitor cellular metabolic states [1, 2].

Fluorescence lifetime image microscopy (FLIM) is an imaging technique that produces an image containing the fluorescence lifetime information of a fluorescent sample at every pixel, that is, an array of pixels with its respective exponential decay rates [3]. FLIM can provide important information about different processes in living cells, but it is mostly used to supervise the metabolic states of a cell [1, 4].

FLIM has been proven useful in biophysics and medical diagnosis research, and it is an ideal approach to study protein–protein interaction [5, 6]. It has been shown that nanoparticles (NPs) have facilitated advances in different fields through the great influence they have in materials due to their physical and chemical unique properties and so in their several applications, such as catalysis, imaging, medical applications, energy-based research, and environmental applications [7, 8]. One of the most relevant properties of NPs is their evaluation using fluorescence lifetime imaging, in living cells for medical research, due to the capability of providing information not only from the biological sample, but from the nanostructured inorganic materials these NPs are made of, including rare earths [9–12].

The phasor plot is a graphical representation of the time delay of the FLIM image in a (*G, S*) coordinate system. Every pixel in the image is converted to a point in the phasor plot. In the same way, every point in the phasor plot has its equivalent pixel in the original image [13, 14]. The phasor method can be used with data from both the time and frequency domains. In this work, data in the frequency domain are used and converted to the phasor plane using the equations (1) and (2),

(1) $$G_{i,j}=M_{i,j}\text{cos}\left(\frac{\pi \;\phi_{i,j}}{180}\right)$$

(2) $$S_{i,j}=M_{i,j}\text{sin}\left(\frac{\pi \;\phi_{i,j}}{180}\right)$$

where, *M_i,j_* is modulation and *ϕ_i,j_* is the phase angle in degrees at each pixel, converted to radians by multiplication with *π*/180, *G_i,j_* and *S_i,j_* are the *X* and *Y* coordinates of the phasor plot, respectively.

The points in the phasor plot can be categorized and therefore segmented based on the fluorescence lifetime of each molecular species in the FLIM image. Segmentation of FLIM intensity and FLIM lifetime-map images is done manually and the results are user-dependent, which can lead to poorly selected clusters and therefore to an inaccurate segmentation of the image [15].

Furthermore, automatic phasor-based segmentation approaches have been previously reported. For example, Zhang *et al* [15] introduced *K*-means clustering directly in the phasor space for intravital FLIM image segmentation, while Vallmitjana *et al* [16] explored multiple machine learning clustering techniques for phasor-based image segmentation. These studies demonstrated that unsupervised clustering can effectively classify phasor distributions without manual cursor selection. However, these approaches heavily rely on user-defined or conventional segmentation methods for background removal and intensity thresholding prior to clustering analysis. Thus, the reproducibility of clustering results depend on the initial thresholding and segmentation preprocessing step.

In the present work, we introduce an imagewise adaptive intensity masking strategy based on kernel density estimation (KDE) and derivative analysis. This preprocessing step is integrated into a complete end-to-end workflow that standardizes background removal prior to phasor clustering. In addition, the method is evaluated in the context of multifunctionalized NP-cell FLIM datasets, where intensity heterogeneity and overlapping phasor distributions pose additional analytical challenges.

## 2. Methods

### 2.1. Multifunctional NPs properties

Gd_2.99_Ce_0.01_Al_1.995_Cr_0.005_Ga_3_O_12_ (GAGG:CeCr) NPs were synthesized through a modified surfactant aided hydrothermal synthesis approach, as indicated in [17]. Chemicals were obtained from Alfa Aesar as well as Fisher Scientific. These NPs were surface-functionalized with folic acid (FA) via aminopropyl trimethoxy silane (APTMS) and FA was covalently conjugated to surface NH2 groups of GAGG:CeCr-NH2 through carbodiimide coupling reaction as described elsewhere [18].

Structural, morphological, electrical and optical properties of multifunctional GAGG:CeCr NPs were characterized in order to evaluate the wide potential applications in imaging techniques. The analysis by XRD revealed a cubic structure in according to probability density function (PDF): 46–0448 crystallographic chart with 34 nm the mean crystallite size and 12.29° A the lattice constant.

The NPs were characterized with no regular shape with an average particle size distribution of 99 ± 46 nm along with well-defined crystal lattice. The importance of NP size is that it lies in the size range that is reported for long-lasting circulation half-life in the body [19].

By means of UV–Vis absorption spectra the presence of Ce^3+^ as well as Gd^3+^ was demonstrated in addition to Cr^3+^ in NPs. As previously known the Ce^3+^ and Cr^3+^ a pair favors the NIR-to-NIR two-photon excited bioimaging despite tissue interference [18].

Additionally, GAGG:CeCr NPs possess paramagnetic properties suitable for magnetic resonance imaging applications with high magnetic susceptibility. The targeting efficiency of the functionalized nanoprobes was assessed through FA functionalization. Cellular internalization of the functionalized nanoprobes was also evaluated, while preserving the core luminescence properties. Excellent biocompatibility of both GAGG:CeCr and GAGG:CeCr-FA NPs was demonstrated in cancer cell lines.

#### 2.1.1. Cell culture

HeLa cells were cultured at 37 °C with 5% CO2 in Dulbecco’s Modified Eagle Medium (DMEM) (high glucos*e) (GeneClone, 25–501 N) su*pplemented with 10% Fetal Bovine Serum (GenClone, 25–514), and 1% penicillin streptomycin (100I U ml^−1^) (Gibco, 15 140 122). The culture was transferred to a flask where the old DMEM medium was extracted, then washed with Dulbecco’s Phosphate Buffered Saline, DPBS (Sigma-Aldrich, 59 331 C-1000ML), lastly, two ml of trypsin (Gibco, 25200056) were added to the culture and incubated for two minutes. Fresh DMEM medium was added after trypsinization and cells were plated in 35 mm glass bottom dishes (1 ml). 1 ml of GAGG:CeCr-FA suspension (1 mg ml^−1^) in DMEM was added to the media, and the cells were incubated at 37 °C with 5% CO2 for 17 h.

The stable human neuroblastoma cell line SH-SY5Y (ATCC) was maintained in culture in Nunc EasYFlask T-75 (Thermofisher, 156472) flasks using cell medium DMEM/Ham’s F-12 (GenClone, 25–503) supplemented with L-Glutamine, 15 mM HEPES, 10% Fetal Bovine Serum, heat Inactivated (GenClone, 25–514 H), and, an antibiotic cocktail of Zeocin 100 *μ*g ml^−1^ (Thermo Fisher, R25005), and Geneticin 120 *μ*g ml^−1^ (Gibco, 10 131 035). Cells were passed every third day or when confluency reached 80% by washing the culture with Dulbecco’s PBS without Ca, Mg, Phenol Red (GenClone, 25–508) and de-attached, with Trypsin-EDTA (0.25%) phenol red (Gibco, 25 200 056). Cultures were split 1:4 every 2 d. Cultures were always maintained at 37 °C in 5% CO2 at saturated humidity. For experiments, a cell density of ~30 000 cells per cm^2^ were plated onto 8-well chambered cover glass dishes.

### 2.2. Additional fluorescent probes

External fluorescent probes used in this work were prepared and used as follows. Early endosomes and recycling vesicles were stained using FM1-43 (Thermofisher T3163), prepared as a 5 mM stock solution per the manufacturer’s instructions and applied at a 1:1000 dilution. All incubations were conducted at 37 °C in 5% CO_2_ under saturated humidity. Green labeled amyloid peptides Beta-Amyloid (1–42), HiLyte^™^ Fluor 488 (Anaspec, AS-60 479-01), was prepared as a stock solution at 2 mM following the providers instructions, and cells were incubated with a final concentration of 400 nM and incubated for 6–8 h before imaging. Cells were always maintained in 5% CO2 incubation conditions.

### 2.3. Confocal and fluorescence lifetime imaging

Fluorescence lifetime images of HeLa and SH-SY5Y cells were acquired on a Zeiss LSM710 (Carl Zeiss, Jena, Germany). Using a 63x oil immersion objective, NA 1.4. Image size is 256 × 256 pixels, pixel *dwell* time of 25.21 *μ*s. Pixel size is ~176 nm. The microscope system is coupled with a two-photon Ti: Sapphire laser (Spectra-Physics MaiTai, Mountain View, CA) with an 80 MHz repetition rate and the samples were excited at 800 nm for HeLa cells and 740 nm for SH-SY5Y cells, at an average power of 5 mW.

A beam splitter 690+ nm was employed to separate excitation from emission signal, followed by a bandpass emission filter 470/22 nm coupled to a photomultiplier tube (PMT) (H7422P-40, Hamamatsu, Japan) were used as the microscope external detector photo-sensor unit to acquire FLIM images. The laser excitation power and detector gain were kept constant for all measurements. Images of Coumarin-6 in ethanol were taken for reference and calibration for FLIM measurements. FLIM data was acquired using an A320 FastFLIM FLIMbox (ISS, Champaign, IL). SimFCS software (www.lfd.uci.edu ) was used for time domain FLIM data acquisition, image referencing and manual image processing.

Confocal images for HeLa cells were obtained with the instrument described above and the following imaging parameters. Image size of 512 × 512 pixels, 16-bit depth, pixel size of ~0.088 *μ*m, pixel dwell time of 0.79 *μ*s, unidirectional scanning, and an averaging method of one frame. The pinhole size was set to its maximum, 599 *μ*m, digital gain to 1, digital offset to 0, and master gain for the PMT detector to 940. For fluorescence collection a 690+ beam filter was used to block light sourcing from the laser, and one channel for the collection of fluorescence emission was set at the range 508–608 nm for green channel. A transmission channel was used with master gain of 100. Excitation was done using a two-photon Ti: Sapphire laser (Spectra-Physics MaiTai, Mountain View, CA) with an 80 MHz repetition rate and the samples were excited at 800 nm at 7% laser power with an average power of 5 mW.

For confocal images of NPs, the all-imaging parameters were kept as in HeLa cells, but the pixel size was decreased to ~0.022 *μ*m, and pixel dwell time of 0.64 *μ*s. A total of 3 field of views (FOVs) with at least one NP were acquired.

Confocal images to visualize exogenous fluorescent molecules in SH-SY5Y cells were carried as follows. Images were acquired using the same instrument described above, with the following acquisition parameters. Image size of 512 × 512 pixels, 16-bit depth, pixel size of ~0.132 *μ*m, pixel dwell time of 2.05 *μ*s, unidirectional scanning, and an averaging method of one frame. The pinhole size was set to 45 *μ*m, digital gain to 1, digital offset to 0, and master gain for all PMT detectors to 700. For fluorescence collection a 488 beam splitter was used to redirect light into two channels for collection of fluorescence emission, these were set at ranges 493–534 nm for green channel and 560–706 for red channel. A transmission channel was used with master gain of 100. A total of five randomly selected FOVs per condition were imaged, with multiple cells in the FOV. A*β* (1–42)–488 and FM1–43 were excited with a 488 nm single-wavelength Argon-Ion laser (LASOS, Jena, Germany) at 3% laser power.

For FLIM and confocal measurements for HeLa cells a total of 25 FOV were collected for cells with NPs, five FOV showing NPs only and five FOVs with only HeLa cells.

Four random FOVs with multiple cells were collected for each experimental condition in SH-SY5Y cells, condition one is A*β*(1–42)–488 and FM1-43, a second condition is FM1-43 only, for both FLIM and confocal images.

#### 2.3.1. Computational procedure

The original FBD files containing lifetime and intensity data were referenced using SimFCS to generate an R64 file comprising three matrices. Specifically, the first matrix contains intensity data from photon arrival into the detector, the second and third matrices convey pixel-wise modulation and phase information, in accordance with equations (1) and (2), which lead to the *G* and *S* images, respectively, and are used to plot as pixel-phasor-coordinates in the phasor plot. Each matrix has dimensions of 256 × 256 pixels. Intensity thresholding and segmentation is done using the first matrix, the FLIM intensity image, while phasor clustering is achieved using the second and third matrices, phase and modulation images.

#### 2.3.2. PDF of an intensity histogram

For 8-bit images, the intensity histogram is a graph that represents the distribution of pixel intensities in a digital image containing 256 possible intensity levels. By utilizing 256 bins, a one-to-one correspondence is ensured between each bin and its respective intensity level. If a smaller number of bins were to be employed, distinct intensities would be grouped together, thereby resulting in a loss of detail.

In FLIM intensity images, pixels consist of floating-point values. These intensities can generally be treated as continuous variables within the specific range of each image. Because choosing the correct number of bins is critical for an adequate histogram representation, performing an analysis based on discrete binning can be inaccurate. Consequently, a superior approach for such continuous variables is to generate a PDF directly from the intensity data [20, 21].

The PDF *f*(*x*) may be derived from the distribution of pixel intensities by treating the image intensity as a random variable. The PDF has to satisfy two conditions: Firstly, *f*(*x*) has to be non-negative; secondly, *f*(*x*) has to integrate to 1, yielding the probability of *X* also equal 1, that is:

(3) $$\int_{\left(-\infty \right)}^{\infty }f(x)dx=1.$$

#### 2.3.3. KDE thresholding for intensity images

KDE stands as a non-parametric method facilitating the approximation of the PDF of a stochastic variable with a limited set of observations or data points [3–6, 22]. For the estimation of a continuous PDF from floating-point intensities, the application of KDE is preferred over traditional binning methods. In this approach, a kernel (commonly a Gaussian function) is assigned to each data point, avoiding the loss of information associated with discrete histogram representations [20].

The PDF, is then estimated mathematically expressed as in equation (4):

(4) $$\overset{ˆ}{f}(x)=\frac{1}{nh}\sum_{i=1}^{n}k\left(\frac{X_{i}-x}{h}\right)$$

where:

*X_i_* is the individual pixel intensity values.

*x* are values from *X_i_*

*n* is the number of the data.

*h* is the bandwidth, which determines the smoothness of the final curve.

*K* is the kernel function, the ‘shape’ used to smooth the point.

In the application of a Gaussian kernel, the aggregate PDF is derived from the summation of individual normal distributions, each centered at an observed data point. The kernels *K* have the form of the standard normal distribution (equations (5) and (6)):

(5) $$K\left(\frac{x-X_{i}}{h}\right)=\frac{1}{\sqrt{2\pi }}\text{exp}\left(-\frac{{\left(x-X_{i}\right)}^{2}}{2h^{2}}\right)$$

then:

(6) $$\overset{ˆ}{f}(x,h)=\frac{1}{n}\sum_{i=1}^{n}\frac{1}{\sqrt{\left(2\pi \right)}h}\text{exp}\left(-\frac{{\left(x-X_{i}\right)}^{2}}{2h^{2}}\right).$$

In this equation, *h* can be interpreted as the standard deviation *σ*.

#### 2.3.4. Bandwidth estimation of the KDE

The bandwidth (*h*) is the most critical parameter in KDE, as the smoothness of the resulting PDF is directly managed by its value (figure 1(A)).

In a small bandwidth regime (Low *h*), each Gaussian component is characterized by low variance and high peak amplitude. While fine details are captured, this often leads to overfitting, where the resulting curve exhibits sensitivity to stochastic noise and appears fragmented (figure 1(A), red line). On the other hand, in a large bandwidth regime (High *h*), each Gaussian component is characterized by high variance and a flattened profile. Although a smoother distribution is produced, underfitting may occur, causing distinct features, such as multimodal peaks, to be obscured or merged into a single distribution (figure 1(A), blue line).

An additional advantage of the KDE method is the mitigation of the inherent sampling noise associated with traditional histograms. Provided that an appropriate bandwidth (*h*) is selected, the discrete fluctuations of the raw data are superseded by a continuous smoothing function, which provides a more statistically representation of the underlying intensity distribution (figure 1(A), green line).

To minimize the mean integrated squared error, several automatic methods can be implemented to determine the optimal bandwidth [21, 23–25]. Computationally efficient heuristics, such as Silverman’s and Scott’s rules, are among the most commonly employed; however, while effective for data approximating a normal distribution, they may induce oversmoothing when applied to more complex distributional structures. A more data-driven alternative is cross-validation, where least squares cross-validation optimizes *h* based on the characteristics of the actual sample. Finally, adaptive bandwidth methods adjust *h* locally, prescribing a smaller bandwidth in high-density regions to preserve fine detail, and a larger one in sparse regions to avoid introducing noise in the distribution’s tails.

FLIM intensity PDFs are considered nonparametric distributions, as the underlying data are derived from physical phenomena characterized by regions of both significantly high and low density. For this specific application, the adaptive bandwidth method is found to be superior to the cross-validation method; it allows for local adjustments to the smoothing parameter, which are required to maintain estimation accuracy across varying density regimes.

In figure 1(B), the intensity map derived from the FLIM intensity data is analyzed to generate its KDE using two distinct algorithms. We show a fixed bandwidth estimated via Silverman’s method is compared against a locally adaptive approach based on the two-stage Abramson estimator [26, 27]. While Silverman’s rule provides a computationally efficient global smoothing parameter, the Abramson estimator allows for the preservation of high-frequency intensity features through local bandwidth modulation. However, these advantages are offset by a higher computational cost and the inherent complexity of its underlying mathematical framework.

The primary objective for segmentation within the FLIM intensity maps is the determination of the optimal threshold between stochastic noise and valid signal. This threshold is localized at the point of maximum probability density within the background distribution. To achieve high computational efficiency in this application, the KDE is constrained to the intensity range containing the highest data density; subsequently, a localized bandwidth is derived via Silverman’s method to define the threshold precisely. By isolating the background intensity regime prior to estimation, the Silverman bandwidth remains highly sensitive to the local noise statistics without being biased by the high-intensity features of the biological sample.

#### 2.3.5. Finding the threshold

The global maximum of the estimated KDE corresponds to the background noise intensity regime, whereas subsequent local maxima represent the dominant intensity populations of the biological sample. To facilitate the separation of the background distribution from discrete signal-bearing populations, a standard approach in image analysis is employed: derivative analysis of the PDF [28, 29].

The optimal transition point between noise and signal is analytically determined through the identification of the local minima of the first derivative and the zero-crossings of the second derivative. This differential approach provides enhanced precision compared to the conventional identification of the global minimum between PDF peaks, as it accounts for the underlying curvature and inflection points of the distribution.

This point represents the maximum rate of decay in the noise population, providing a mathematically deterministic boundary between the stochastic background and the biological signal.

While KDE is theoretically defined as a continuous function, its computational implementation yields a discrete numerical set corresponding to the pixel count of the image. Consequently, the rate of change within this discretized framework is most effectively computed using the gradient function (equation (7)),

(7) $$f^{\prime }\left(x\right)=\frac{f\left(x+\Delta x\right)-f\left(x-\Delta x\right)}{2h}+O\left((\Delta x{)}^{2}\right)$$

where:

$f^{\prime }(x)$ is the first derivative of the function *f* at point *x*

*f*(*x* + Δ*x*) *and f* (*x* − Δ*x*) are function values at a small distance Δ*x* ahead of and behind the point of interest.

$O\left((\Delta x{)}^{2}\right)$ is the truncation error, proportional to the square of the step size Δ*x*.

The numerical implementation of the discrete gradient utilizes central differences rather than forward difference approximations. This approach is mathematically superior for identifying slope transitions within intensity distributions, as it provides second-order accuracy $O\left((\Delta x{)}^{2}\right)$ [30] and effectively minimizes the phase shift in the resulting derivative. Consequently, the local minima and inflection points, critical for defining the background threshold, are localized with significantly higher precision.

This threshold is determined directly from the statistical structure of the intensity distribution, ensuring a reproducible and data-driven selection without user intervention.

### 2.4. Thresholding comparison with other thresholding algorithms

We used six widely used automatic thresholding algorithms available in the Python library skimage.filters to calculate threshold values for each image in each dataset for comparison with our KDE thresholding approach. The algorithms were Li, Mean, Otsu, Triangle, Yen, Isodata. The calculated threshold value is then applied to the FLIM intensity image followed by a quantification of the total number of pixels greater than zero, corresponding to the foreground signal, the resulting value represents the total-pixel-area, and is used for comparison against the other thresholding methods and a proxy ground truth (GT).

To set a GT for each image, we used a user-defined threshold value determined by a FLIM-expert. The threshold value was then applied as described above to obtain the GT total-pixel-area, which is then used for thresholding validation against the other methods.

Thus, the thresholding validation employs an expert proxy and area-based metric where we compare the total pixel area between the GT and every thresholding method, we use the ratio of the total-pixel-area of the thresholding method divided by the GT total-pixel-area: ratio = ThrMethod/ThrGT. This ratio represents how larger or smaller the thresholded area is compared to the GT area. A value of 1 indicates perfect agreement with the GT, values greater than 1 indicate overestimation, and values lower than 1 indicate underestimation.

### 2.5. Clustering of phasors

Cluster analysis applies practical procedures to obtain a classification. Clustering algorithms include a variety of unsupervised techniques for finding patterns or groups (clusters) within a set of observations. These algorithms are based on a statistical procedure to organize a collection of data into homogeneous groups with some measure of similarity between them and presenting a difference with the data grouped in other clusters [31].

One of the metrics used to measure or quantify the similarity between two sets of data is the Jaccard Index, developed by Paul Jaccard [32], defined by equation (8):

(8) $$J(A,B)=\frac{|A\cap B|}{|A\cup B|}$$

where *A* and *B* are two sets of data respectively, *J* (*A, B*) represents the Jaccard index that can take values between zero and one, where zero indicates no similarity, and one indicates complete similarity. This index is the relationship between the intersection size between both sets ∣*A*∩*B*∣ and the size of the union, respectively ∣*A*∪*B*∣.

The calculation of the Jaccard similarity coefficient requires two sets of data to make the comparison; this value can also be represented as a percentage. In this work, six clustering algorithms were used to cluster phasors in every image in the dataset: Gaussian mixture model (GMM), *K*-means, Mini Batch *K*-Means, Bayesian Gaussian mixture (BGM), Agglomerative clustering, and Balanced Iterative Reducing and Clustering using Hierarchies (BIRCH). Three paradigms were used before applying each algorithm, these paradigms are applied to the *G* and *S* images, corresponding to the module and phase, respectively, of the recorded lifetime measurements associated to each pixel in the FLIM intensity image: with no filter, application of the median filter with a 3 × 3 neighborhood and application of the median filter with a 5 × 5 neighborhood.

### 2.6. Statistical analyses

Statistical analysis was performed using GraphPad Prism (www.graphpad.com) and we report mean with standard error mean (SEM).

## 3. Results

### 3.1. KDE implementation for FLIM intensity images

The FLIM Intensity image, often referred to as the total intensity image, is the integrated photon count per pixel over the total acquisition time across the entire recorded decay time range.

In FLIM images, the photon count range can vary significantly based on sample brightness, laser excitation power, and the sensitivity of the detectors, such as PMTs or hybrid detectors [33]. Most biological FLIM experiments utilize a dynamic range of 100–1000 photons per pixel. Within the range of 0–50 photons per pixel, the signal becomes indistinguishable from background noise or detector ‘dark counts’ [34]. Zero to ten (0–10) photons is usually considered pure noise. Whereas 10–50 photons is considered a Low-Count regime. Although basic spatial structures remain visible in the intensity domain, the photon statistics within this range are insufficient for reliable lifetime estimation. It is standard practice to threshold out these pixels to maintain the integrity of the resulting lifetime maps.

The intensity histogram facilitates the visualization of the statistical separation between the biological sample and background noise. The background peak, typically appearing as a prominent spike near zero, comprises detector dark counts and ambient electronic noise; in contrast, the signal distribution appears as a broader curve or shoulder to the right. The threshold in a FLIM intensity histogram is a specific cutoff value that determines which pixels are included in the lifetime analysis (figure 2).

A common practice in intensity histogram analysis is the identification of the *local minimum* separating these two populations. However, establishing the threshold at this point does not necessarily ensure the total exclusion of noise, nor does it fully preserve the integrity of the biological signal in low-count regimes. Manual thresholding based on histogram inspection allows for subjective optimization; however, this iterative process is inherently non-reproducible and difficult to implement in automated workflows (figures 2(A)–(E)).

If the threshold is set too low, background noise is included (figure 2(C)). Conversely, if the threshold is too high, dim but biologically relevant signals may be discarded (figure 2(E)).

FLIM images contain inherent intensity variations across a dataset, where each image has its own local range of intensity values, meaning background cannot be defined by a fixed intensity value universally applied to all images. Each FLIM image is a 256 × 256 pixel array with real-valued intensities; for example, figure 2(A) spans a range from 0 to 12.397 intensity levels, as shown in its corresponding histogram with 180 bins. Because the distribution of intensity values is non-uniform across bins, the histogram can present several zero-count bins, as visible in the right-hand side region (figure 2(G)).

The separation of stochastic background noise from the biological signal is achieved by calculating an optimal intensity threshold from the raw FLIM intensity data through a staged algorithm. First, fluorescence and lifetime data are extracted from the referenced files, retaining the raw intensity values as the primary observation set (figure 2(A)). An intensity distribution analysis is then performed, where the photon count distribution in the intensity histogram is analyzed to identify a global maximum representing the most frequent intensity regime, typically associated with the background noise floor. For instance, the FLIM intensity image (figure 2(A)) and its respective histogram (figure 2(G)) show a concentration of low-intensity values, with a maximum frequency centered at an intensity value of 0.265; the adjacent intensity values comprise the background range to be removed.

Because the background and sample intensities are often very similar, a direct histogram-based threshold can be insufficient to differentiate them (figures 2(A)–(E)). To extract a smooth, continuous profile of the intensity distribution, KDE is applied. KDE is a well-established smoothing technique that builds an approximation function capturing only fundamental patterns while filtering irrelevant noise. Applying KDE to a FLIM image of 65 536 pixels is equivalent to constructing a PDF of the sum of 65 536 Gaussians. Within the localized range near the global maximum, a Silverman bandwidth is derived to establish an appropriate smoothing scale. The resulting KDE curve, illustrated as the green density curve in figure 2(H), is a smoothed continuous representation of the intensity histogram.

However, the KDE function alone does not provide a clear intensity transition point between background and signal. Therefore, the first-order derivative of the KDE is computed via a second-order accurate central-difference scheme, which enables high-fidelity visualization of the distribution’s slope; this is shown as the blue curve in figure 2(H). A local minimum in this derivative corresponds to an inflection point in the KDE function and identifies a candidate threshold value. The first local minimum, situated immediately adjacent to the primary noise peak, is selected as the optimal segmentation threshold, since it corresponds to the intensity closest to the dark background region (figure 2(H), magenta line). For example, this approach yields a threshold value of 0.496 for the original FLIM intensity image (figure 2(F)).

This threshold is then applied pixel-wise: all pixels with intensities below the determined value are systematically excluded, producing a segmented intensity map that retains only valid biological signal for subsequent lifetime analysis. This KDE approach streamlines the transition from a stochastic intensity distribution to a digitally segmented morphology, demonstrating the efficacy of the derivative-based thresholding in isolating biological structures from the background noise floor.

### 3.2. Comparison of thresholding with KDE vs other algorithms

Global standard tools for bio-image analysis, like ImageJ/Fiji [35] continue to offer classical segmentation methods because they are fast, require no training data, and can yield highly reproducible results for high-contrast samples when properly used. Some of the most common thresholding methods used to separate foreground from background are Otsu, Li, Yen, and Phansalkar [36, 37]. For edge detection, it is common to use filters like Canny or Sobel to identify boundaries [38].

To evaluate the effectiveness of our thresholding method on FLIM intensity images, we computed the total segmented pixel area resulting from applying different thresholding methods across five datasets representing distinct contrast conditions: a single high-contrast agent where only NPs are present (figure 3(A)), a single low-contrast agent corresponding to dim autofluorescence of NADH in cells (figure 3(B)), a combination of low- and high-contrast agents where cells and NPs are found in the same FOV (figure 3(C)), multiple high-contrast agents show cells with two bright exogenous fluorophores A*β*(1–42) peptides labeled with green fluorophore HLyte488 and orange fluorescence molecule FM1-43 labeling recycling vesicles and early endosomes (figure 3(D)), and multiple low-contrast agents where cell autofluorescence and FM1-43 signal mixes (figure 3(E)). The classification of a fluorescent molecule as a high- or low-contrast agent was determined by its quantum yield, a property reflecting the efficiency of light emission following photon absorption, alongside the overall photon count observed in the respective intensity histograms. Figure 3 presents the spatial distribution of these agents and their fluorescence emission collected from one- or two-channel confocal microscopy images, confirming the presence of single or multiple contrast agents in each dataset.

Working directly on the FLIM intensity data, to obtain a GT thresholding value, a FLIM expert sets an user-defined threshold value, which is then compared against six common automatic thresholding methods; mean, triangle, li, Isodata, Otsu and Yen. The minimum thresholding method is not reported in this work as it resulted in multiple calculation errors for the majority of images across all datasets. The threshold values were separately applied to the same FLIM intensity image to remove the background signal, followed by calculating the total-pixel-area in pixel units for each newly created masked image (figure 4).

*Single high contrast* images correspond to very bright fluorescent NPs imaged in solution (figure 4(A)). *Single low contrast* condition corresponds to imaging the autofluorescence of NADH, a dim molecule as seen by its intensity histogram, in HeLa cells (figure 4(B)). *Low and high contrast* are images containing fluorescence of NPs, bright, and NADH, dim inside HeLa cells in the same detection channel when excited at 800 nm with 2-photon (figure 4(C)). *Multiple variable contrast* images correspond to images of SH-SY5Y cells with two exogenous fluorescent molecules, A*β*(1–42) peptides tagged with HiLyte-488 are bright and FM1-43 signal is dim at 740 nm with 2-photon excitation, and the endogenous biomolecule FADH also dim under same conditions, the fluorescence emission of all three was collected in the same detection channel (figure 4(D)). In the *multiple low contrast* analysis, SH-SY5Y cells in images contain two very dim fluorescent molecules, FM1-43 and FADH (figure 4(E)). Noteworthy to mention that NADH and FAD autofluorescence is very dim to sometimes detect with traditional confocal imaging parameters without compromising cell viability, only FLIM measurements and their photon counting capabilities are suitable to detect and record their weak autofluorescence at wavelengths above 740 nm with 2-photon excitation.

Visual results indicate that our KDE thresholding approach produces segmentations that show good agreement with those generated by the FLIM expert. However, this agreement is contingent on a sufficiently large range of intensity values in the histogram, that is, the KDE method yields segmentations consistent with the GT only when the signal-to-noise ratio (SNR) is large enough to produce a well-separated intensity distribution. (Figure 4, manual and KDE columns).

Table 1 summarizes the area ratios between the different thresholding methods and (GT) across varying signal-to-noise conditions. On average, the KDE thresholding method described in this work yields threshold values closely consistent with the GT, provided the SNR is sufficient to produce a clear separation between background and signal in the intensity histogram. In low-contrast or noisy images, however, where this separation is not well-defined, the method fails to identify a reliable threshold for background removal. This limitation is inherent to the method’s reliance on a heterogeneous intensity distribution: the KDE approach requires sufficient variability in both intensity values and their frequencies to accurately localize the largest change in the histogram, a condition that is not met when background and signal intensities are poorly differentiated. This limitation is explicitly considered when interpreting segmentation performance.

### 3.3. Phasors clustering after image segmentation

Pixel-level phasor coordinates are affected by photoncounting noise and local intensity fluctuations, which can introduce dispersion in the phasor distribution and affect clustering stability. As previously described in phasor-based FLIM analysis protocols [14], moderate spatial smoothing can improve cluster coherence without altering the intrinsic lifetime information.

Thus, median filtering was selected instead of linear averaging filters because it preserves spatial boundaries and reduces impulsive noise while minimizing distortion of phasor distributions. The filter was applied after background removal and prior to clustering in order to stabilize the spatial organization of phasor pixels while maintaining the underlying lifetime structure. Applying two very common paradigms of spatial smoothing, we evaluated the robustness of clustering algorithms under controlled spatial smoothing conditions. Median filtering was applied independently to both *G* and *S* matrices using 3 × 3 and 5 × 5 neighborhoods.

In the work of Vallmitjana *et al* [16], different grouping algorithms were used when obtaining the Jaccard index. According to the values obtained, GMM is the best grouping and comparison method since the phasor data generally presents a normal dispersion, it provides better results even when overlapping data exists. Thus, we decided to set GMM as our proxy-ground-truth (pGT) for phasor clustering.

GMM, *K*-means and some of the other unsupervised clustering methods require the number of clusters *k* to be defined in advance. In this workflow, the number of clusters *k* is an expert-defined parameter rather than an automatically calculated value. This approach leverages valuable domain knowledge, as the biological context and experimental conditions in FLIM provide critical guidance that rigid mathematical criteria might overlook. Based on the empirical observation that the phasor distributions consistently exhibited a small number of visually distinct clusters, *k* was defined by the user to ensure the segmentation strictly aligns with the underlying biochemical components. As illustrated in figure 5, different experimental conditions produce distinct phasor distributions: undisturbed NADH in cells appears as a single cluster (figure 5(A)), NPs in solution occupy a well-defined region (figure 5(B)), and when both are present in the same FOV, multiple phasor populations emerge (figure 5(C)). Under these conditions, three clusters were typically identified, associated with NP signal, undisturbed cellular NADH metabolism, and NADH metabolism perturbed by NP internalization. This selection was applied consistently across methods to enable comparability between clustering approaches.

The presence of a NP within a cell perturbs its local metabolic activity, shifting the free-to-bound NADH ratio, while neighboring cells and their metabolic signatures remain largely unaffected. Simultaneously, the fluorescence lifetime signature of the NP may also shift due to two main factors: the mixing of multiple lifetimes within pixels occupied by the NP, and the altered intracellular physicochemical environment, which can inherently modify fluorescence lifetime properties (figures 5(C) and (D)) [39, 40].

In summary, the number of clusters *k* is associated with the dominant contrast sources observed in the sample. In this work, three main clusters were consistently identified: NPs, undisturbed NADH metabolism, and NADH metabolism perturbed by NP internalization. It is important to note that NADH metabolism itself reflects a continuous distribution driven by the free-to-bound NADH ratio (figure 5(d), magenta circles).

To validate our KDE thresholding within a full FLIM analysis pipeline, we applied it to the Low + High Contrast experimental dataset described above, which contains images of HeLa cells exposed to functionalized NPs. This experimental condition produces heterogeneous intensity histograms in which conventional thresholding algorithms struggle to preserve dim fluorescence signal that nonetheless carries relevant biological information (figure 4(C) and table 1, Low + High Contrast column). Figure 6 presents a representative summary of the clustering outcomes for a single image, where KDE thresholding is first applied to the FLIM intensity image, followed by a spatial median filter on the *G* and *S* images, prior to phasor clustering with each method.

Since GMM is considered the standard approach for phasor clustering, it serves as the reference control for all comparisons using the Jaccard similarity index. Across the majority of images in the dataset, all median filter conditions and clustering methods successfully assigned spatial labels to the three phasor clusters, though with substantial differences in the number of phasors assigned to each cluster (figure 6, top row).

Table 2 summarizes the pairwise Jaccard comparisons across the three spatial filter conditions for every clustering method. These comparisons are made through agreement analysis between algorithms using GMM as the baseline reference. Without any filter applied, *K*-means achieved the highest median similarity to GMM at 0.57, while BIRCH showed the lowest at 0.45. With a 3 × 3 median filter, *K*-means again yielded the highest similarity (median = 0.71), and BGM the lowest (median = 0.48). With a 5 × 5 median filter, *K*-means reached the highest median similarity of 0.83, while BIRCH remained the lowest at 0.48.

The progressive increase in Jaccard similarity with larger filter kernels reflects improved spatial coherence of phasor coordinates rather than artificial inflation of clustering agreement. Pixel-level noise can fragment clusters in the phasor plane, and median filtering suppresses this stochastic variability while preserving intrinsic lifetime characteristics. The 3 × 3 kernel offered a favorable balance between noise suppression and structural preservation, while the 5 × 5 kernel further improved cluster stability at the cost of mild spatial homogenization (figure 6). Importantly, these comparisons reflect the relative agreement with the chosen reference algorithm (GMM) rather than clustering accuracy against an independent GT. Consequently, while moderate spatial filtering enhances clustering agreement and stability, further validation is required to confirm whether these distributions correspond to actual biological or lifetime components.

### 3.4. Reliable and automatic phasors segmentation

In conventional phasor-FLIM analysis workflows, cluster selection and intensity thresholding are typically performed interactively by the user, requiring manual cursor placement in the phasor plot and visual identification of intensity boundaries (figure 6, Original column) [14]. As described in established phasor protocols, this process depends on the operator’s interpretation of phasor cloud distributions and may introduce variability between users, particularly when adjacent clusters partially overlap.

The present method proposes an automated approach that removes the need for manual threshold selection and phasor cursor positioning by applying a data-driven intensity mask, obtained with a KDE-based thresholding algorithm, followed by unsupervised clustering. This automatic workflow produces segmentation patterns that show agreement with manual analysis under suitable imaging conditions while reducing user dependency and providing computationally efficient processing, only and only if the FLIM intensity image contains enough recorded photons and the imaging system has been optimized to maximize the SNR.

## 4. Discussion

### 4.1. FLIM intensity images segmentation applying KDE

The proposed algorithm provides a reproducible and mathematically deterministic framework for the automated segmentation of FLIM intensity maps. By transitioning from traditional histogram-based thresholding to a KDE approach, the stochastic noise and discretization errors inherent to raw photoncounting data are mitigated, addressing a known limitation of conventional thresholding approaches. Optimizing the imaging system to maximize the SNR is essential for the KDE approach to perform correctly. In images with low contrast or poor SNR, this method tends to overestimate the threshold value (figure 4, table 1).

The core strength of this methodology lies in its data-driven localization of the background-to-signal transition. Using a second-order accurate centraldifference gradient, the algorithm identifies the local minimum of the first-order KDE derivative immediately adjacent to the primary noise peak. This point represents the candidate transition point of the background distribution decay, providing a consistent segmentation boundary that minimizes the heuristic bias associated with manual threshold selection. The strategic use of a localized Silverman bandwidth further ensures computational efficiency without compromising the structural integrity of the biological signal, enabling effective separation of data populations even in low-photon regimes, a critical requirement for accurate fluorescence lifetime estimation.

In practice, the KDE method provides a reproducible and image-specific thresholding strategy for segmenting cells within the FOV. In contrast to manual segmentation, which requires interactive user-defined threshold selection, the proposed approach applies the same statistical criterion across images, thereby reducing user dependency and improving standardization of the segmentation process. Ultimately, this algorithm supports segmentation of biologically relevant signal and provides a standardized tool for quantitative analysis of fluorescence microscopy data in fluorescence microscopy.

### 4.2. Phasor map clustering with ML algorithms

Once segmented *G* and *S* images are obtained via KDE-based thresholding, median filtering is applied prior to phasor clustering using six unsupervised algorithms: *K*-means, Mini Batch *K*-Means, GMM, BGM, Agglomerative clustering, and BIRCH [7–11].

These algorithms differ meaningfully across four key dimensions: computational efficiency, cluster shape flexibility, parameter sensitivity, and interpretability. In terms of efficiency, *K*-means and Mini Batch *K*-Means are the most scalable, with Mini Batch *K*-Means offering additional speed gains through random data subsampling. GMM and BGM are more computationally demanding due to probabilistic parameter estimation, particularly in highdimensional spaces. Agglomerative clustering carries quadratic time complexity, which can become prohibitive for large datasets, while BIRCH, though designed for scalability, manages memory more efficiently through compact data summarization.

Regarding cluster shape, *K*-means, Mini Batch*K*-Means, and GMM assume convex, isotropic clusters, which may limit their performance on complex or non-linear distributions. Agglomerative Clustering and BIRCH are more flexible in accommodating arbitrary cluster shapes through hierarchical structuring and local density information. BGM further extends this flexibility by incorporating a Bayesian framework that can infer the number of components automatically. In terms of parameter sensitivity, all methods require some form of prior specification, *K*-means and Mini Batch *K*-Means require the number of clusters *k* to be defined in advance, as do GMM and BGM, though their probabilistic formulations make them more robust under uncertainty. Agglomerative Clustering is sensitive to the choice of linkage criterion and distance metric, while BIRCH requires tuning of the merging threshold and maximum cluster count. Finally, in terms of interpretability, *K*-means and Mini Batch *K*-Means provide straightforward hard cluster assignments, whereas GMM and BGM yield probabilistic memberships that better capture assignment uncertainty. Agglomerative Clustering and BIRCH produce hierarchical structures that can reveal inter-cluster relationships, albeit at the cost of more complex interpretation.

While previous studies have demonstrated the feasibility of automatic clustering in phasor space [15, 16], the present work emphasizes the importance of automated intensity preprocessing prior to clustering. By integrating KDE-based adaptive masking with unsupervised phasor clustering, the proposed workflow reduces dependence on manual threshold selection and improves consistency across heterogeneous FLIM datasets. The primary contribution of this study therefore lies in the image-specific preprocessing strategy and its integration with established clustering techniques, rather than in the clustering algorithms themselves.

### 4.3. Comparison with AI-based segmentation tools

Recent advancements in FLIM have increasingly leveraged artificial intelligence to move beyond traditional intensity thresholding [41–45]. Commercial microscopy platforms such as Zeiss Intellesis and Leica Aivia, among others, now incorporate supervised deep learning modules that offer powerful segmentation capabilities for intensity-based and morphological image analysis. However, these systems typically require user-provided training datasets, manual annotation for model calibration, and iterative parameter tuning, meaning that performance is contingent on the quality and representativeness of the training data, and generalization across diverse datasets can vary considerably.

Precise threshold detection nevertheless remains critical in FLIM analysis, as it enables discrimination between background noise and true fluorescence signal, even in low-photon regimes, and existing thresholding algorithms vary considerably in their ability to differentiate signal from background across different sample types.

In contrast, the workflow proposed here is fully unsupervised and requires no annotated training data. The KDE-based threshold is determined in a data-driven, image-adaptive manner, and the subsequent clustering step operates directly in phasor space, preserving the physics-based lifetime representation central to phasor-FLIM analysis [13, 14]. Because each computational step is explicitly defined, the approach is inherently transparent, reproducible, and interpretable. Furthermore, the entire pipeline can be implemented using standard open-source scientific computing tools, without proprietary software licenses or specialized hardware, facilitating broader accessibility in academic settings.

The intent of this work is therefore not to compete with deep learning-based segmentation methods, but to offer a transparent, interpretable, and cost-accessible alternative tailored specifically to the characteristics of phasor-FLIM datasets.

## 5. Conclusions

Traditional lifetime analysis using the Phasor approach requires interactive threshold adjustment and cursor selection in the phasor plot, processes that depend on user interpretation and experience. While experienced operators can achieve consistent results, the procedure inherently involves subjective decision-making steps that may introduce variability across users and datasets.

In this work, an automated workflow eliminates manual parameter selection, enabling more standardized processing of multiple images under identical computational criteria. This method provides in datasets with enough contrast between background and foreground signal, this method provides a reproducible and user-independent segmentation strategy obtained by applying the KDE method and its derivative. This way, the intensity threshold value is automatically calculated, and a binary mask is generated that allows the background of the FLIM image to be removed. As a result, a segmented image is obtained and contains the area of interest to analyze.

The choice of clustering algorithm hinges on various factors including computational resources, data characteristics, interpretability requirements, and the specific objectives of the analysis. Evaluating these algorithms against these considerations and conducting empirical assessments on specific datasets are paramount in determining the most suitable approach for a given clustering task.

## Figures and Tables

**Figure 1.**
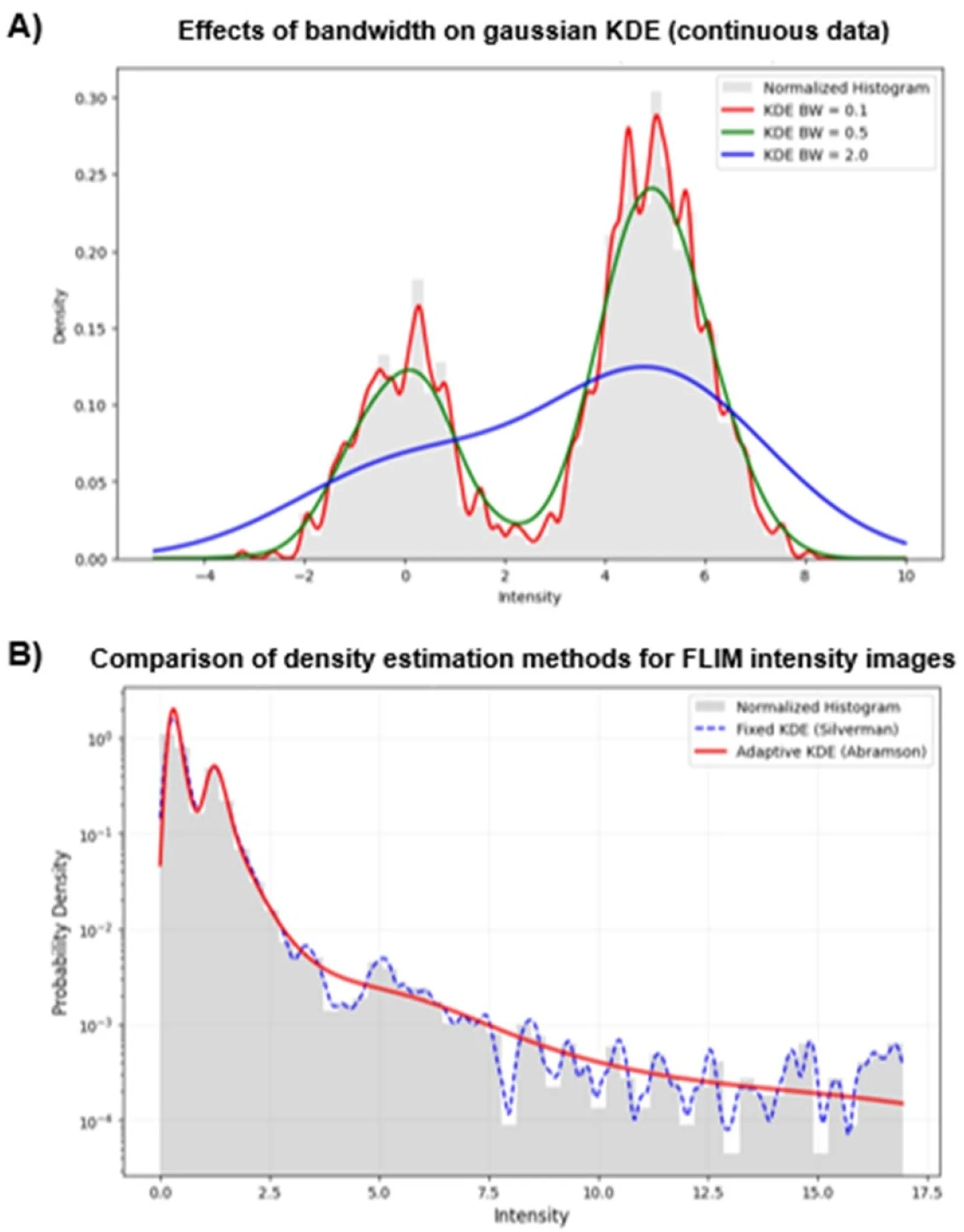
Kernel density estimation selection. (A) Impact of bandwidth selection on Gaussian kernel density estimation (KDE). A comparative analysis of a randomly generated bimodal distribution is performed using three discrete bandwidths: *h* = 0.1, 0.5, 2.0. (B) The probability density functions (PDFs) are shown for two different smoothing approaches. The fixed bandwidth, optimized via Silverman method (blue line), provides a global smoothing parameter, whereas the adaptive method (red line) locally adjusts the bandwidth *h* to accommodate the high dynamic range.

**Figure 2.**
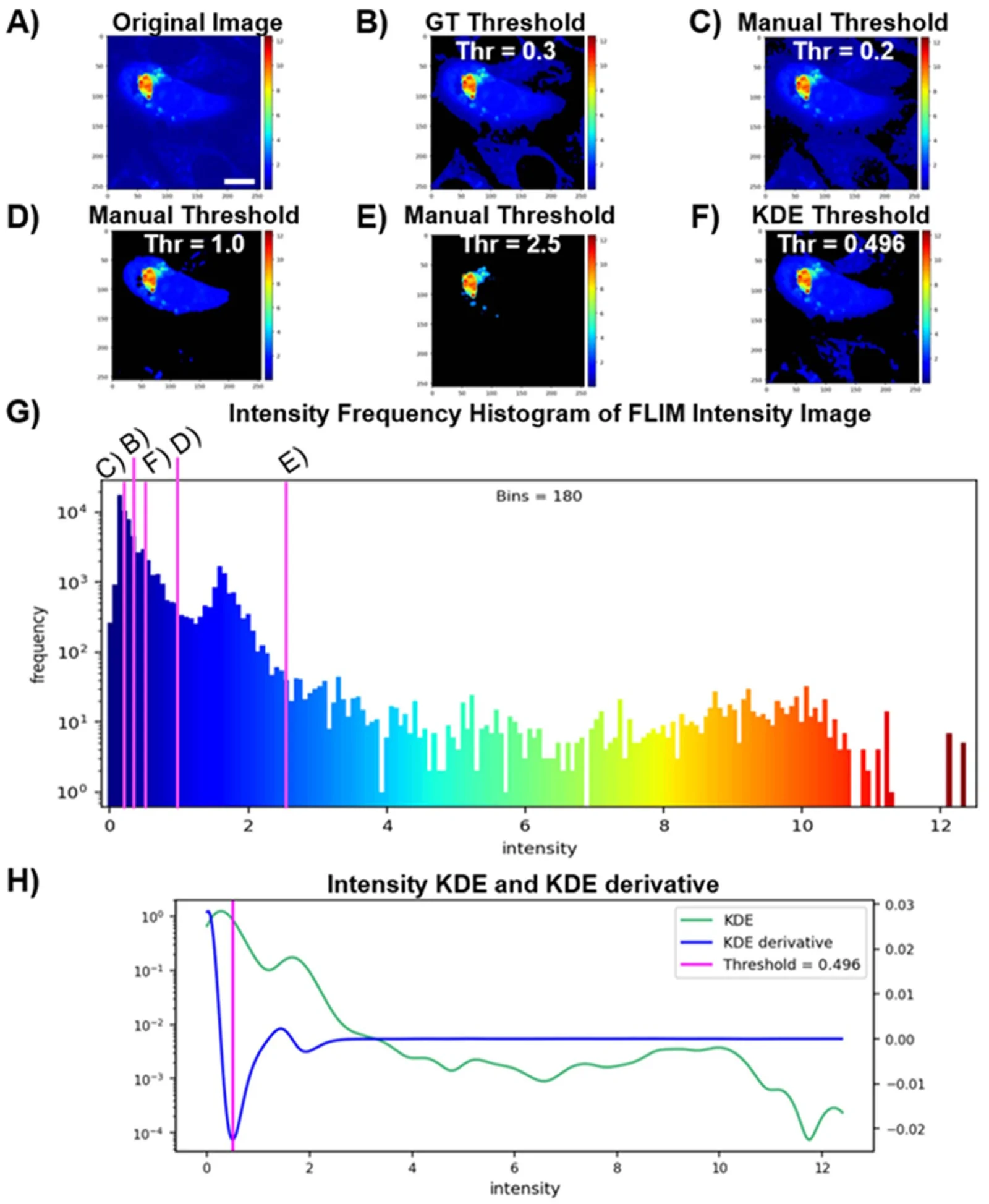
FLIM intensity image with manual thresholding options. (A) FLIM intensity image in pseudocolor. (B) Threshold value (Thr) selected by a FLIM expert and used as ground truth (GT). (C) Underestimation of the Thr value; segmentation includes background signal. (D) Overestimation of the Thr value; segmentation eliminates signal from dim cells in the field of view. (E) Overestimation of the Thr value; segmentation removes most cellular signal and retains primarily nanoparticle (NP) signal. (F) Intensity histogram of the FLIM intensity image. (G) Intensity histogram with five manually selected threshold values (magenta lines) illustrating different background subtraction outcomes. (H) Probability density function (PDF) obtained via KDE. The green curve represents the KDE, the blue curve its first derivative, and the magenta line indicates the automatically determined threshold corresponding to the first local minimum of the derivative.

**Figure 3.**
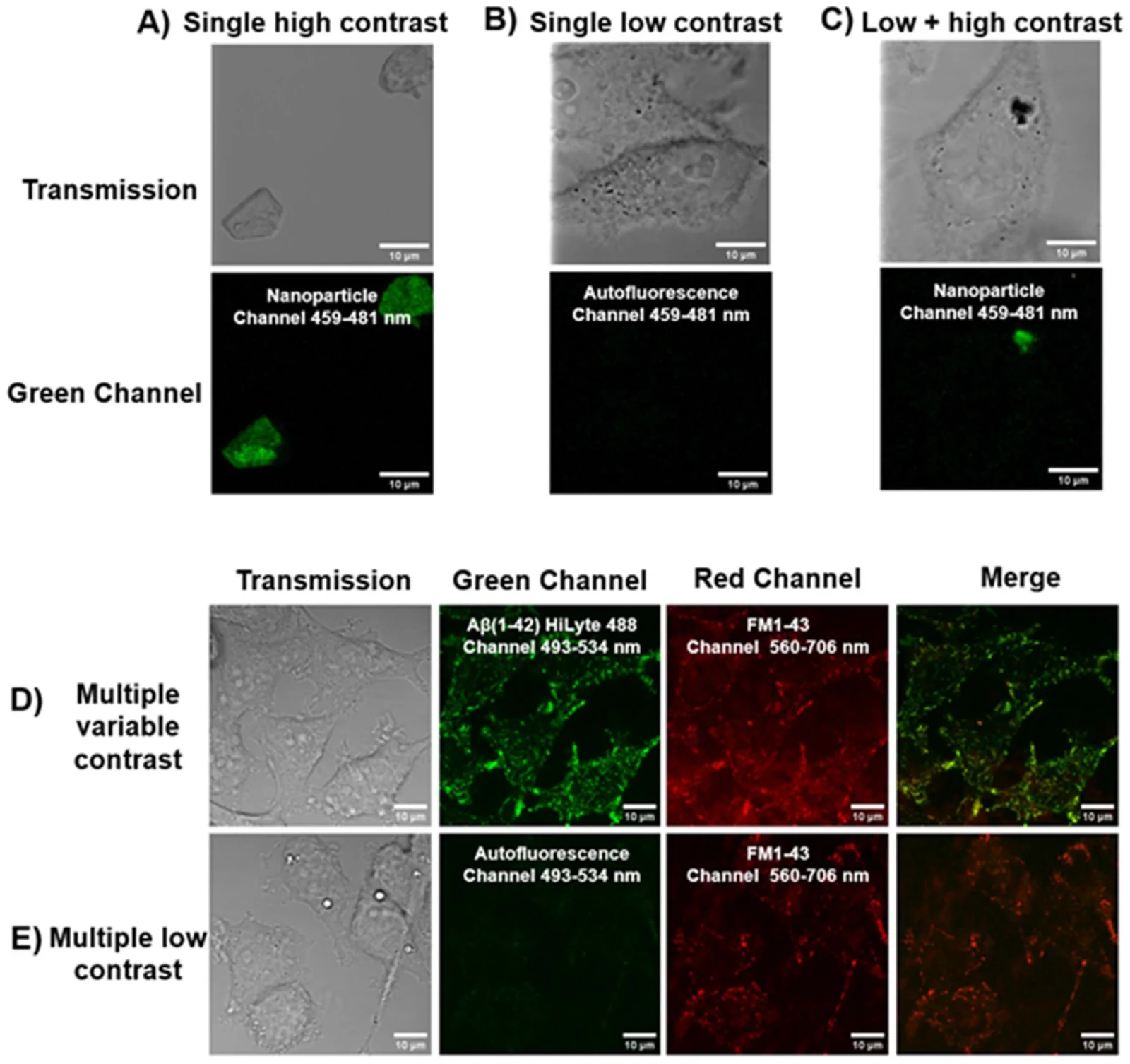
Representative images of different contrast conditions. In top panel, the top row corresponds to transmission images, and the bottom row is the fluorescence collected in the green channel for (A) single high contrast with only nanoparticles in the field of view (FOV), (B) single low contrast of the autofluorescence intensity of NADH(H) in cells, (C) low and high contrast with nanoparticles and NAD(H) fluorescence in cells. In the bottom panel, from left to right, the transmission image, then the fluorescence collected in the green and red channels, followed by the merge of both. The top row correspond to (D) multiple contrast corresponds to the mixture of fluorescent molecules A*β*(1–42)HiLyte488, FM1-43 and FAD(H) autofluorescence in cells, and the bottom row to (E) multiple low contrast corresponds mixture of fluorescent molecules FM1-43 and FAD(H) autofluorescence in cells. The scale bar is 10 *μ*m.

**Figure 4.**
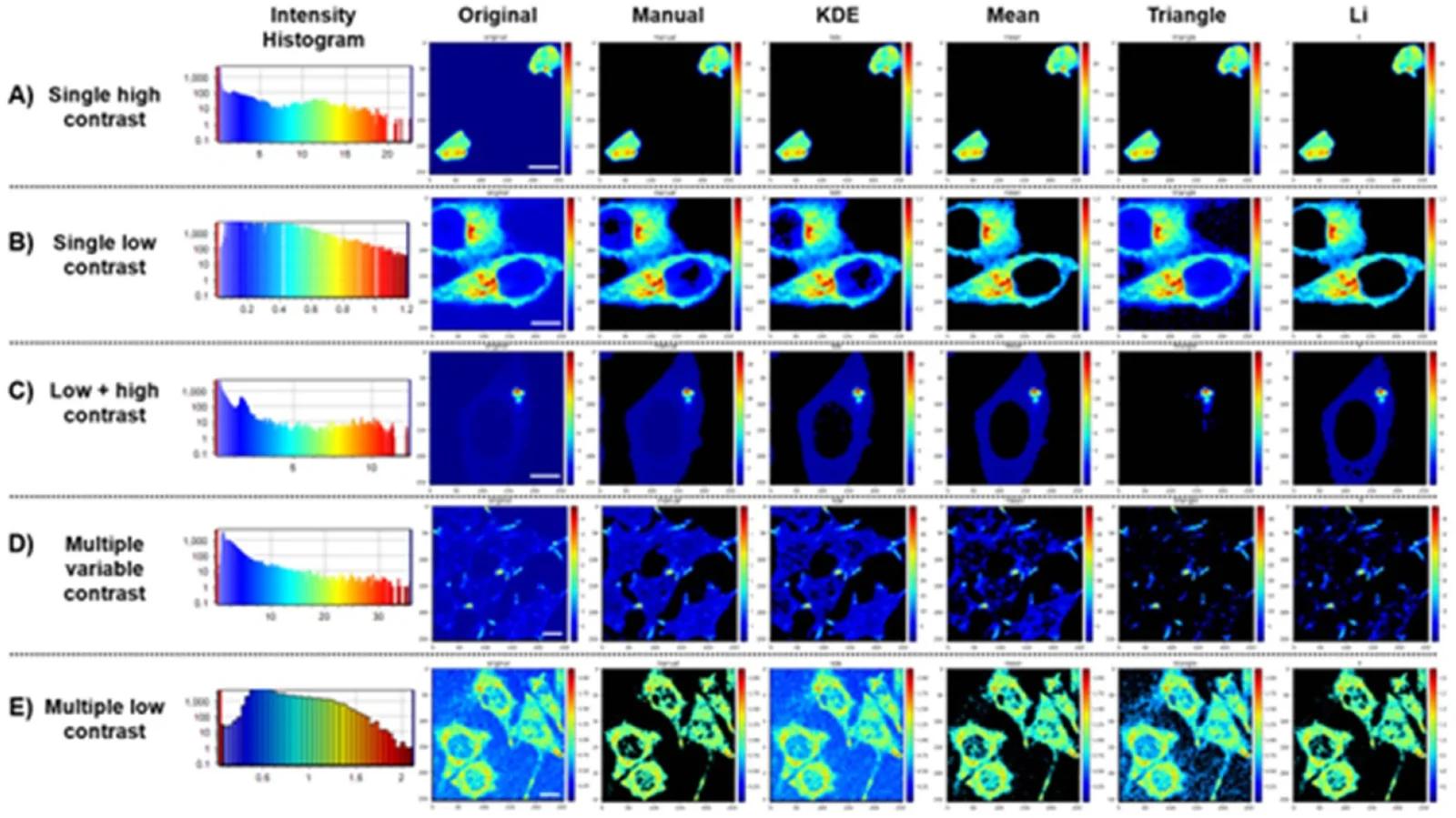
Representative comparisons of different thresholding methods performing when applied to different contrast conditions. Columns from left to right display the intensity histogram and the corresponding original FLIM intensity image, followed by images thresholded with the methods; manual, KDE, mean, triangle and li (see table 1 for a full summary of the thresholding performance). Rows from top to bottom correspond to (A) single high contrast with only nanoparticles in the field of view (FOV), (B) single low contrast of the autofluorescence intensity of NADH in cells, (C) low and high contrast with nanoparticles and NADH fluorescence in cells, (D) multiple contrast corresponds to the mixture of fluorescent molecules A*β*(1–42)HiLyte488, FM1-43 and FAD(H) autofluorescence in cells, and (E) multiple low contrast corresponds mixture of fluorescent molecules FM1-43 and FAD(H) autofluorescence in cells. The scale bar is 10 *μ*m.

**Figure 5.**
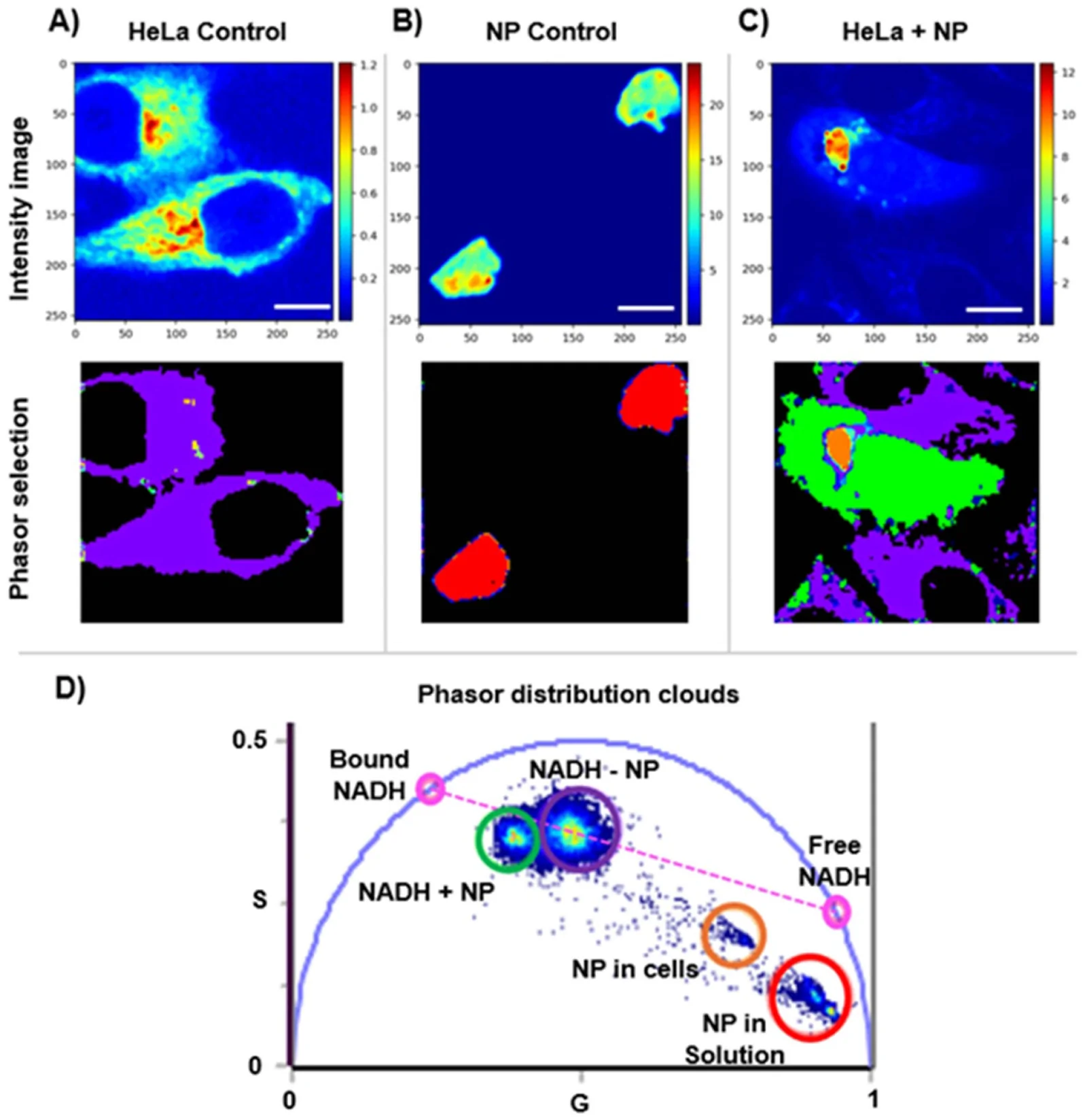
Phasor distributions in HeLa and nanoparticles experimental conditions. (A) FLIM intensity image of HeLa cells control experiment (top) and its respective lifetime-map based on manual selection (bottom). (B) FLIM intensity image of nanoparticles in solution (top) and its respective lifetime-map based on manual selection (bottom). (C) FLIM intensity image of nanoparticles and HeLa cells (top) and its respective lifetime-map based on manual selection (bottom). (D) Phasor plot with phasor distributions corresponding to lifetimes characteristic of different experimental conditions, magenta circles are the free and bound pure locations of NADH autofluorescent molecule, the dashed magenta lines represent the trajectory along which NADH lifetime signatures move across when the ratio of free-to-bound is changed. The purple circle is NADH unaffected by external factors and provides a baseline location of the NADH phasor distribution. Green circle corresponds to affected NADH phasor distribution when NP are present. Red circle corresponds to the characteristic lifetime of NPs in solution. Orange circle corresponds to the NP signature when present inside HeLa cells. Scale bar is 10 *μ*m.

**Figure 6.**
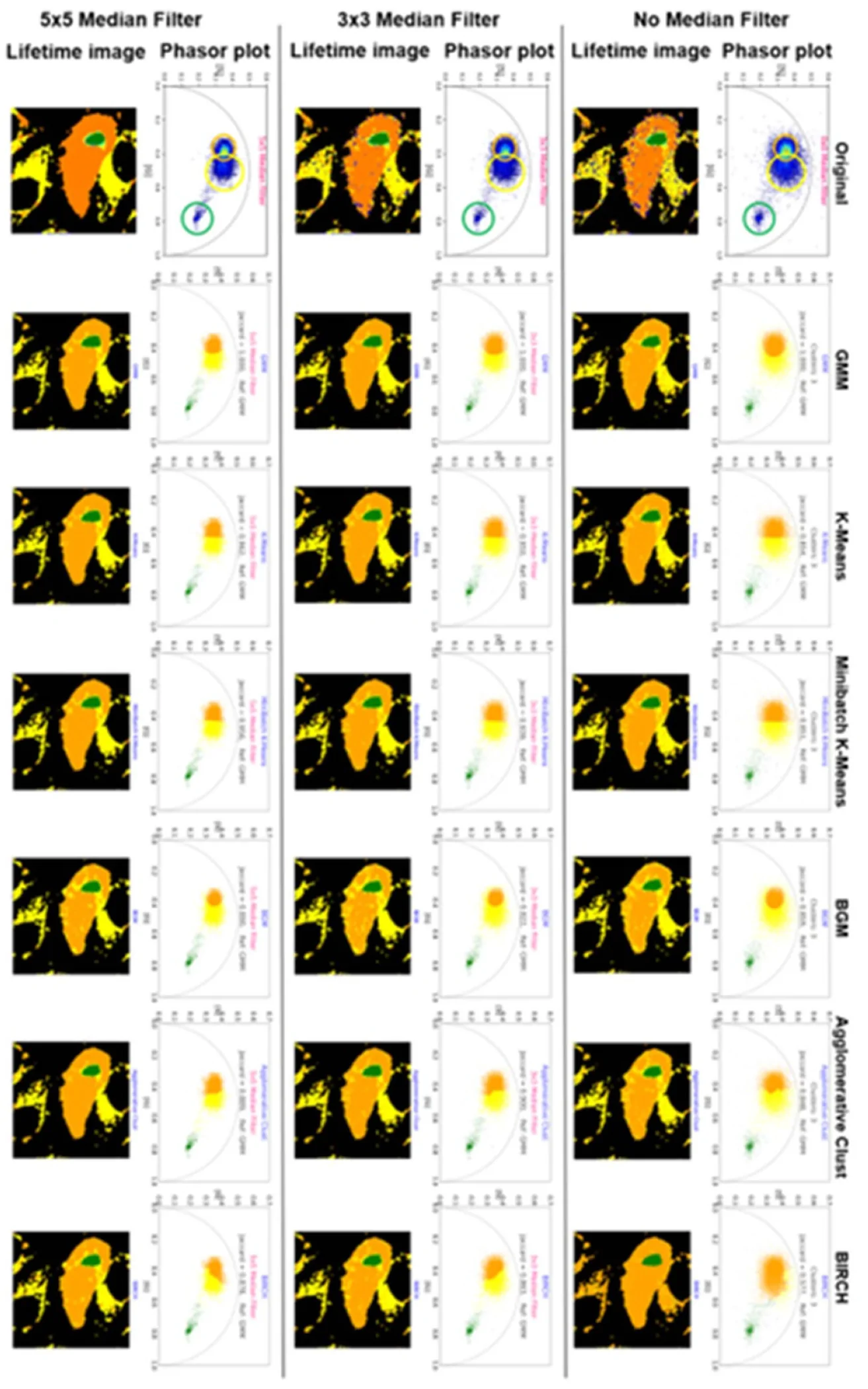
Comparison of phasor clustering with unsupervised models, a phasor plot with clustering highlighted with circles when manual, and color-coded when automated. Clusters are orange, yellow and green with its respective color-coded lifetime image underneath. Figure is rotated 90 degrees clock-wise. From top to bottom, analysis of phasor clustering with (top) no median filter, (middle) 3 × 3 median neighborhood, and (bottom) 5 × 5 median neighborhood filter applied to *G* and *S* images. From left to right, original, GMM, *K*-Means, BGM, agglomerative clust and BIRCH.

**Table 1.** Summary table of the average-difference-ratio values between ground truth (GT) and different thresholding methods applied to different contrast conditions. A value closer to one, 1, represents a closer value to GT. Numbers in bold represent the best performing thresholding algorithm for each experimental condition. Error is expressed as the standard error mean (SEM). The number of analyzed FOVS is represented with *n*.

Thresholding method vs GT	Single high contrast (*n* = 4)	Single low contrast (*n* = 14)	Low + high contrast (*n* = 27)	Multiple high contrast (*n* = 5)	Multiple low contrast (*n* = 5)
GT	1	1	1	1	1
KDE	**0.88 ± 0.25**	**1.03 ± 0.12**	**0.90 ± 0.34**	**0.91 ± 0.06**	3.43 ± 1.64
Li	0.67 ± 0.38	0.73 ± 0.12	0.54 ± 0.35	0.30 ± 0.11	**1.02 ± 0.12**
Mean	1.18 ± 0.45	0.75 ± 0.11	0.81 ± 0.13	0.60 ± 0.04	1.21 ± 0.20
Otsu	0.53 ± 0.32	0.49 ± 0.19	0.27 ± 0.32	0.04 ± 0.01	0.76 ± 0.12
Triangle	0.88 ± 0.41	1.09 ± 0.54	0.64 ± 0.65	0.01 ± 0.06	2.26 ± 0.66
Yen	0.76 ± 0.44	0.20 ± 0.19	0.07 ± 0.17	0.06 ± 0.03	0.46 ± 0.42
Isodata	7.71 ± 14.06	0.49 ± 0.20	0.39 ± 0.31	0.03 ± 0.02	0.85 ± 0.15

**Table 2.** Jaccard coefficient summary table for low + high contrast dataset, HeLa cells with functionalized NPs. Results correspond to the clustering of phasors on three different sizes of median filters paradigms, no median filter applied, 3 × 3 median neighborhood and, 5 × 5 median neighborhood applied to the *G* and *S* images before clustering. Values are expressed as the median (25% percentile/75% percentile). The number of FOVs analyzed are represented as *n*.

Algorithm	Jaccard similarity coefficient (J)		
	No filter (*n* = 27)	Median filter 3 × 3 (*n* = 27)	Median filter 5 × 5 (*n* = 27)
GMM	1.0	1.0	1.0
*K*-Means	0.57 (0.45/0.83)	0.71 (0.27/0.91)	0.83 (0.55/0.89)
Mini batch *K* -Means	0.48 (0.43/0.74)	0.55 (0.42/0.84)	0.68 (0.45/0.84)
BGM	0.45 (0.36/0.65)	0.48 (0.39/0.66)	0.49 (0.43/0.68)
Agglomerative clustering	0.51 (0.36/0.64)	0.62 (0.42/0.76)	0.58 (0.35/0.75)
BIRCH	0.45 (0.35/0.57)	0.5 (0.33/0.69)	0.48 (0.36/0.63)

## Data Availability

All data that support the findings of this study are included within the article (and any supplementary files).
